# Varifocal MEMS mirrors for high-speed axial focus scanning: a review

**DOI:** 10.1038/s41378-022-00481-0

**Published:** 2023-10-27

**Authors:** Jaka Pribošek, Markus Bainschab, Takashi Sasaki

**Affiliations:** Silicon Austria Labs, Villach, Austria

**Keywords:** Micro-optics, Optical materials and structures

## Abstract

Recent advances brought the performance of MEMS-based varifocal mirrors to levels comparable to conventional ultra-high-speed focusing devices. Varifocal mirrors are becoming capable of high axial resolution exceeding 300 resolvable planes, can achieve microsecond response times, continuous operation above several hundred kHz, and can be designed to combine focusing with lateral steering in a single-chip device. This survey summarizes the past 50 years of scientific progress in varifocal MEMS mirrors, providing the most comprehensive study in this field to date. We introduce a novel figure of merit for varifocal mirrors on the basis of which we evaluate and compare nearly all reported devices from the literature. At the forefront of this review is the analysis of the advantages and shortcomings of various actuation technologies, as well as a systematic study of methods reported to enhance the focusing performance in terms of speed, resolution, and shape fidelity. We believe this analysis will fuel the future technological development of next-generation varifocal mirrors reaching the axial resolution of 1000 resolvable planes.

## Graphical abstract

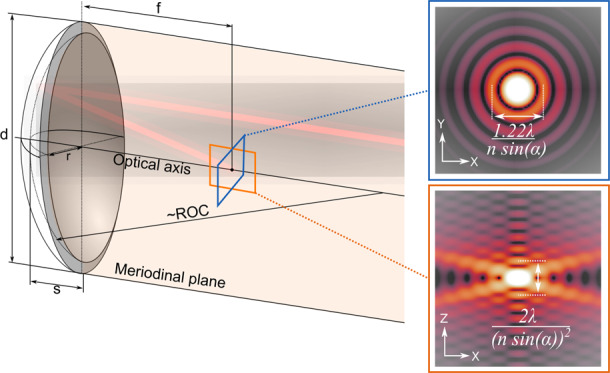

## Introduction

Many modern applications rely on fast scanning of the focal point in three dimensions. Prominent examples include concurrent signaling monitoring of several hundreds of neurons across connected brain areas^1–3^, studying subcellular dynamics^4^, resolving the vergence-accommodation conflict in 3D augmented reality projections^5^, or femtosecond laser processing of curved surfaces^6^. While lateral scanning can easily achieve microsecond response time and resolutions in excess of several thousand resolvable points, scanning in an axial direction presents a bottleneck in systems’ throughput, speed, and resolution. Electro-optical ceramic lenses, transient acoustic gradient lenses, liquid crystal lenses, tunable fluidic lenses, as well as alternative remote focusing techniques^7^ constitute the main state-of-the-art technologies for fast focus control, extensively reviewed in ref. ^8^.

Varifocal MEMS mirrors were shown to be able to control higher-order aberrations^9^, achieve high axial resolutions exceeding 300 resolvable planes^10,11^, can achieve 10 μs response times^12^, continuous operation speeds above several hundred kHz^13^, and can be designed to combine lateral steering with focusing in a single chip-sized device^14^. The fact that varifocal MEMS mirrors can be produced with cost-effective semiconductor microfabrication makes them particularly attractive for consumer electronics where scalability is a strict requirement. As such, varifocal MEMS mirrors are holding promise to replace other varifocal technologies in a variety of fields soon.

Varifocal mirrors have been actively researched in the last 50 years, gaining additional momentum since the early 2000s. A variety of different actuation mechanisms have been employed to control the shape of the reflective membranes and the way performance-relevant properties are reported varies from research group to research group. The resulting heterogeneous set of available data complicates the comparison of the results achieved so far and also obscures the vision of the technological paths to be taken to meet future challenges. With the introduction of a new figure of merit, this work proposes a concept to unify the way the performance of varifocal MEMS mirrors is reported. The figure of merit enables a fair performance comparison of mirrors of different geometries and actuation principles. Based on the analysis of more than 80 mirrors reported in the literature, this work represents the first comprehensive survey of varifocal MEMS mirrors, and assesses the limitations and advantages of different technologies and specific solutions to improve their performance in speed, resolution, and shape fidelity. Future directions to unveil their full potential are discussed.

## Fundamentals

**Fig. 1:**
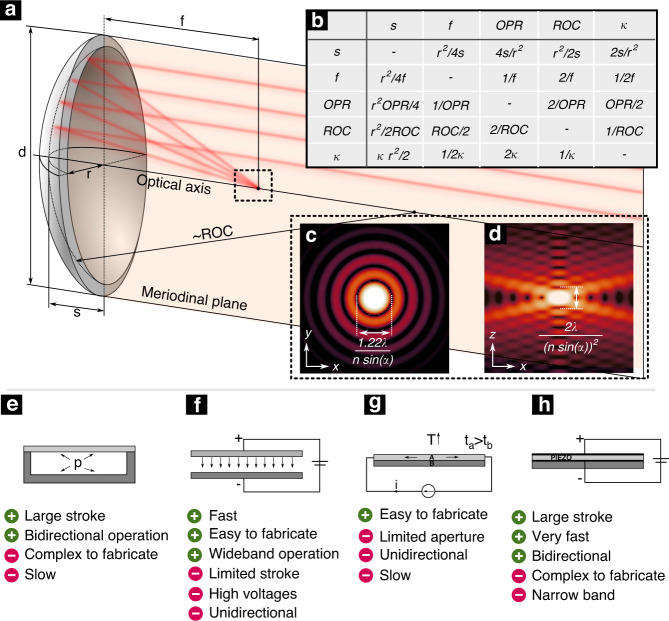
Working principle of varifocal MEMS mirrors. **a** Schematic illustration of a varifocal MEMS mirror and its fundamental geometric and optical properties. **b** Relationships between the geometric and optical properties in a tabulated form. **c** Lateral point spread function. **d** Axial point spread function. **e** Pneumatic actuation, **f**. Electrostatic actuation, **g** Thermal actuation, **h** Piezo actuation

Varifocal mirrors are reflective optical elements with tunable curvature, used to control the axial position of the focal spot. Figure 1 shows an actuated varifocal mirror with a circular membrane. The corresponding geometrical parameters *d* and *r* are the diameters and the radius of the membrane. The optical power is given by OPR and the radius of curvature is by ROC. The curvature is described as *κ*. The mirror’s sag is approximated as $s(r)=2f+\sqrt{4{f}^{2}-{r}^{2}}$ with *f* being the focal length. The relationships between all different parameters are derived in the tabulated form in Fig. 1b–d show the lateral and axial point spread function of a Gaussian beam focused by the mirror in the axially symmetric case.

The lower part of Fig. 1 summarizes the different actuation principles, used to control the deformation of the thin reflective membrane: (i) pneumatic and hydraulic actuation, (ii) electrostatic, (iii) thermal, and (iv) piezoelectric actuation. The first reported mirror was actuated pneumatically by a loudspeaker in 1961^15^. The concept got soon adopted by others^16,17^ and is still in use today^18^. Pneumatic actuation relies on controlling the pressure in the sealed fluidic cavity underneath the flexible membrane. This concept brings several advantages: first is the uniform distribution of pressure, independent of the stroke, mirror geometry, or actuator type. Consequently, a pure parabolic deflection without any spherical aberrations can be achieved^19^. The second advantage is that the system’s working point can be shifted freely by adjusting the initial pressure. This allows the deformation of the membrane and the use of actuators with single directional force, as returning force is achieved by the counteraction of the fluid^20^. Compared to pneumatic actuation, hydraulic actuation is much less common, especially due to the increased complexity, associated with filling and sealing the fluid chamber with hydraulic liquid.

The most abundant actuation principle in the literature is, by far, electrostatic actuation. Designed as parallel plate capacitors, these mirrors are built from a flexible conductive membrane placed above the counter electrode. Applying the voltage across the mirrors provides electrostatic pressure attracting the membrane towards the counter electrode.

Thermally actuated varifocal mirrors are made of two layers of materials with different coefficients of thermal expansion. By increasing the temperature of such a stack, for example by leveraging the Joule heating effect, layers expand in different proportions and induce a change in the curvature of the mirror. While these devices are simple to fabricate, the disadvantage includes unidirectional control, limited stroke, and susceptibility to temperature loading in uncontrolled environments.

Piezoelectric mirrors are built as a composite stack of a supporting membrane and a piezoelectric layer. An electric field, applied to the piezoelectric layer induces mechanical strain, resulting in the deformation of the supporting membrane. The comparably high stiffness of the substrate typically limits the static response, but the structures benefit from resonant operation at high frequencies, large generated forces, and general design flexibility of the piezoelectric actuation.

## Comparison of varifocal mirrors

This work compares the performance of more than 80 varifocal mirrors reported in the literature. To provide a foundation for a universal concept to describe the performance of tunable reflective optics, we propose a merit function defined as a combination of axial resolution and the speed of operation. This way, the figure of merit correlates well with the overall system throughput and is analogous to the figures of merit, introduced for lateral scanning systems^21^. The maximum number of resolvable planes *N*_planes_^22^ is a practical way of expressing the axial resolution of an optical system. This expression can be derived directly from the Rayleigh criterion (Appendix B):

1 $${N}_{{\mathrm{planes}}}=\frac{2}{\lambda }{{\Delta }}s$$

with *λ* and Δ*s* being the wavelength and total stroke of the mirror, defined as Δ*s* = *s*_max_ − *s*_min_. Somewhat non-intuitively, the axial resolution does not depend on the mirror diameter, other device parameters, or additional optics but is instead only a function of the total mirror stroke Δ*s*. However, since the stroke is difficult to measure directly, we rather express it as ${{\Delta }}s={d}_{{\mathrm{eff}}}^{2}{{\Delta }}{\mathrm{OPR}}/16$ in terms of total optical power range ΔOPR and effective mirror diameter *d*_eff_, both parameters being simple to measure experimentally. Finally, we introduce the operation frequency of the mirror to define the figure of merit for varifocal mirror:

2 $${\mathrm{FoM}}={{\Delta }}{\mathrm{OPR}}\cdot {d}_{{\mathrm{eff}}}^{2}\cdot f\,[{{{\rm{Hzm}}}}]$$

The rationale for choosing this FoM is to be a simple merit involving parameters simple to measure and thus widely reported in the literature. We note, however, that the proposed figure of merit neglects the possible deviations from the ideal mirror shape that might arise from mirror geometry, its inertia, or the nonlinear stiffening of the mirror suspensions. These shape imperfections give rise to optical aberrations, which broaden the point spread function and thus deteriorate the practically achieved axial resolution. Hence, the number of resolvable points might be, in practice, lower than the one obtained through Equation (1). However, shape fidelity is rarely, if at all, consistently measured and reported. The existing efforts dealing with shape fidelity are discussed in the subsection “Surface shape fidelity enhancement”. In the future, researchers are encouraged to report the shape fidelity or to experimentally characterize the number of maximally resolvable points.

**Fig. 2:**
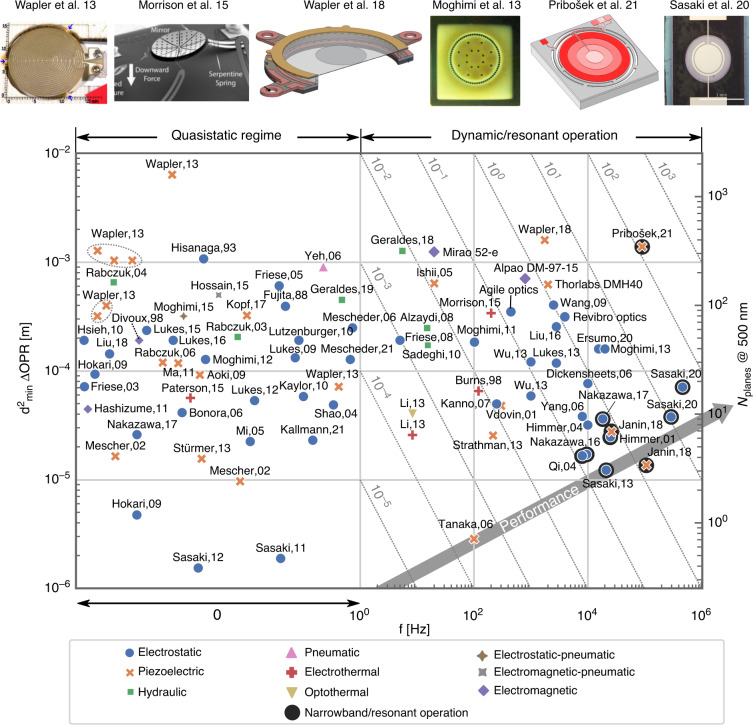
Graph of the figure of merit, plotted as ${d}_{{\mathrm{eff}}}^{2}{{\Delta }}{\mathrm{OPR}}$ vs frequency. Dotted lines represent isolines of a constant figure of merit. The top row shows selected best-performing designs from the literature. The right-hand side denotes the corresponding number of resolvable points, which was derived from the figure of merit and serves as an indication only. Reprinted with permission from [10–13, 32, 39]

Figure 2 shows the mirrors’ operation frequencies *f* and the product of effective membrane diameter squared times the range of optical power (${d}_{{\mathrm{eff}}}^{2}{{\Delta }}{\mathrm{OPR}}$) in a double logarithmic plot. Different marker styles and colors represent the various actuation mechanisms. Mirrors, with only static operation reported, are positioned in the left section of the plot (0 Hz). The diagonal dotted lines represent isolines of a constant figure of merit according to Equation (2). The data represented in Fig. 2 is available in a tabulated form in Table 1. The right-hand side indicates the theoretical number of resolvable planes, calculated for a wavelength of 500 nm and derived from the mirrors’ strokes. Since we did not consider reported shape accuracies, the actually achieved number of resolvable planes is likely lower than shown here. The top row of Fig. 2 shows selected devices featuring the highest performance, which are discussed in detail in the sections further below.

### Comparison between different actuation types

Figure 2 indicates correlations between the mirrors’ performances and actuation principles. Both electrostatically and piezoelectrically actuated mirrors cover very broad ranges of frequencies and ${d}_{{\mathrm{eff}}}^{2}{{\Delta }}{\mathrm{OPR}}$ products. While electrostatic mirrors excel at higher operation frequencies and larger bandwidths, piezo mirrors exhibit the largest ${d}_{{\mathrm{eff}}}^{2}{{\Delta }}{\mathrm{OPR}}$ products in quasistatic and dynamic operations. Hydraulically and thermally actuated mirrors could only be operated in the sub-kHz frequency regime but can reach ${d}_{{\mathrm{eff}}}^{2}{{\Delta }}{\mathrm{OPR}}$ products exceeding 1 × 10^−4^ m. Combined actuation principles or electromagnetic actuation represent only a small fraction of reported devices, some of which will be discussed in the following sections.

### Comparison against other technologies

Varifocal mirrors offer considerable advantages over other existing focus-tuning technologies reviewed recently by ref. ^8^. Compared to various refractive optical elements like mechanically tunable Alvarez lenses and tunable liquid lenses, varifocal mirrors feature roughly four times higher optical power for the same surface sag^22^. As mirrors are built as thin, lightweight, and flexible membranes, an order of magnitude higher-axial resolution and two to three orders of magnitudes faster response compared to liquid lenses can be achieved. A distinct feature of varifocal mirrors is their capability of resonant operation at extremely large deformations, offering a strong alternative in applications where tunable acoustic gradient (TAG) lenses are typically used. Modern varifocal mirrors offer a comparable speed of operation to TAG lenses at a much larger optical power and the ability to control higher-order aberrations besides quadratic profiles. The commercial state-of-the-art TAG lens delivers 0.3 m^−1^ optical power at 11 mm aperture and 70 kHz, yielding ${d}_{{\mathrm{eff}}}^{2}{{\Delta }}{\mathrm{OPR}}$ of ca. 3.6 × 10^−5^ m. The best mirror presented in this review^23^ delivers a figure of merit of over 1 × 10^−3^ m at 90 kHz, which is roughly 30 times better. Compared to refractive optical elements and liquid crystal lenses, varifocal mirrors can focus light independently of their input polarization state, provide significantly higher ablation thresholds, and do not suffer from chromatic aberration or group delay dispersion. The latter is currently a feature, unmatched by any of the existing technology. Another important advantage over alternative technologies is fabrication. While hydraulic and pneumatic mirrors share similarities in fabrication to the liquid-tunable lenses, piezoelectric, thermal and electrostatic fabrication can be designed to be fully compliant with the highly scalable semiconductor microfabrication process.

## Technological improvements of varifocal mirrors

### Optical power and stroke enhancement

**Fig. 3:**
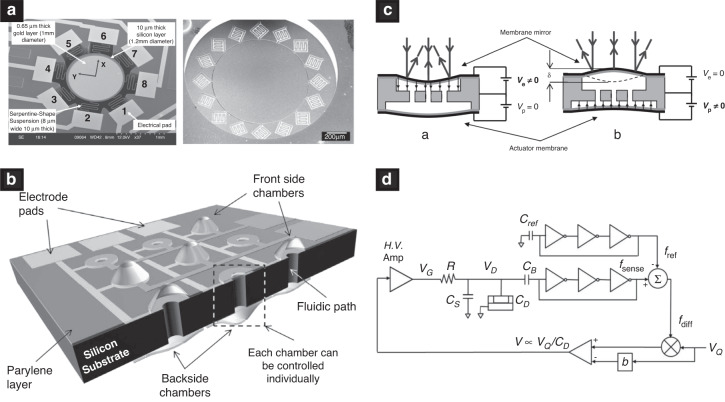
Stroke enhancement approaches. **a** Stroke enhancement by reduction of the support reactions^37^. **b** Stroke enhancement by hydraulic amplification^35^. **c** Bidirectional actuation utilizing a pneumatic link between two unidirectional actuation membranes^34^. **d** Closed-loop control to maximize the stroke to 75% of the air gap^25^. Reprinted with permissions from^25,34,35,37^

As the axial mirror resolution is shown to be purely dependent on the mirror stroke (see section “Fundamentals”), main research efforts have been directed to enhance it. Figure 3 shows different approaches to enhance the stroke of varifocal mirrors. In electrostatic mirrors, the stroke is directly proportional to the electrostatic pressure, which is the reason for the persistent increase of the actuation voltage. Still, two fundamental problems are imposing limits on the maximum stroke in practice: first is the static snap-down effect which occurs at roughly 44% of the air gap^24^ and the second is the electric breakdown which occurs in the air at electric fields above 3 kV mm^−1^. To overcome the snap-down limit, a closed-loop control scheme was proposed to regulate the applied voltage in inverse proportion to the mirror’s capacitance, thus increasing the useful static stroke to 75% of the total air gap^25^. When driven dynamically at the mechanical resonance, successful operation of up to 60% of the air gap was demonstrated. Nevertheless, to enhance the stroke of an electrostatic mirror it is inevitable to increase the air gap and, consequently, the driving voltage. To circumvent this, ref. ^26^ segmented the optical area into several hundred small actuators. This way, the stroke of each actuator was minimized to 550 nm, however when individual actuators are combined, it is possible to achieve 10 μm of total stroke, corresponding to 37.6 theoretically resolvable planes^26^. Another approach to increase the stroke is to reduce the reaction forces of the membrane suspension. Given the circular membrane with certain uniformly distributed pressure, it can be shown that the simply supported mirror reaches (5 + *ν*)(1 − *ν*)/(1 − *ν*^2^) higher displacement than the edge-clamped membrane. Here, *ν* stands for Poisson number. For (100) silicon, this yields an improvement factor between 4.125 to 4.750 for <100> and <110> directions, respectively. This is why a large number of authors devised mechanical features to reduce the bending moment of the membrane suspension. To this end, authors reported several different suspension mechanisms: weak-rim support^27,28^, node support^13^, radial beam suspension^29^, tangential cantilevers^30^, serpentine-shaped springs^27^, compliant mechanisms, and flexures^11^. A large number of these studies were directed to improve the shape fidelity, discussed in the subsection “Surface shape fidelity enhancement”.

Another issue, concerning the stroke in the quasistatic operation regime, is that the force generated by thermal and electrostatic actuators is unidirectional, hence allowing only concave mirror operation. One way to allow both actuation directions with a unidirectional force is by statically preshaping the membrane into a convex shape, deforming it into a concave shape upon actuation. This method was demonstrated for both an electrostatic^31^ as well as an electrothermal mirror^32^ but was found to degrade the mirror shape in the convex and flat operation region. To generate true bidirectional operation, Bonora & Poletto^33^ reported an electrostatic mirror design with a second transparent conductive layer, placed above the mirror membrane to generate the pull-out force. Another way to generate bidirectional operation was found by ref. ^34^ who reported a mirror with two electrostatically actuated membranes, sharing the same fluidic chamber. Due to the pneumatic link between the both, the actuation of one membrane allows a convex operation of the other membrane. Furthermore, this design can be utilized to provide pressure amplification and a resulting increase of the stroke of the optically active membrane, which ref. ^35^ reported in a hydraulic mirror. Hydraulic actuation has the potential to generate large forces and, thus, strong membrane deformations. Geraldes et al.^36^, for example, presented a hydraulic mirror using a silicon nitride membrane and a motorized micro-injection system with 10 kPa to achieve an optical power of 80 m^−1^ corresponding to a stroke of 80 μm.

In thermal mirrors, considerable limitations with respect to stroke apply due to the nature of the actuation principle. The energy required to heat the mirror is proportional to the mass of the membrane; therefore, electrothermal mirrors are typically small devices and consequently require high curvatures to reach useful strokes. With only 0.4 mm diameter ref. ^32^ reported an optical power of 2132 m^−1^ to reach a stroke of 21 μm. In 2013, ref. ^37^ reported a 1.2 mm thermally actuated mirror. They noted a maximum allowable Joule heating power to be 33 mW to avoid thermal damage, limiting the OPR to 18 m^−1^. To further enhance the stroke, optothermal actuation using 488 mW laser was added to boost the optical power range by an additional 10 m^−1^. The same design was later actuated using an external Peltier element to extend the tunable range to 39 m^−1^^38^.

Among the reported piezoelectric mirrors, most of the designs rely on transverse excitation employing the *d*_31_ piezoelectric coefficient. As an alternative ref. ^39^ reported lateral excitation of the lead-zirconium-titanate piezoelectric ceramics using the *d*_33_ coefficient. Since the *d*_33_ coefficient typically is two times larger than the *d*_31_ coefficient, authors were able to achieve a 400 μm stroke in a conical reflective axicon mirror design. This, to date, is the largest stroke ever reported (see the top row in Fig. 2).

### Speed and frequency range enhancement

**Fig. 4:**
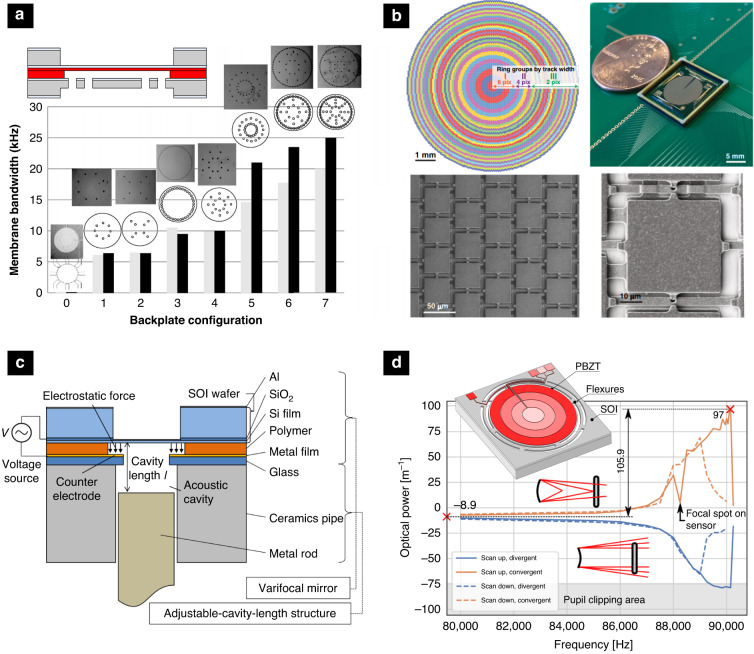
Selected approaches to enhance the operation speed. **a** Transient response enhancement by different perforation patterns^12^. **b** Reducing the viscous damping and increasing the stroke by segmenting the optical area in the several hundred actuators^26^. **c** Increasing the frequency bandwidth by tuning the acoustical cavity^43^. **d** Artificially increasing the frequency bandwidth by engineering geometric nonlinearities^11^. Reprinted with permissions from^11,12,26,43^

The quest for higher system throughput in various machining and measurement applications drives the development toward faster focus shifting in the axial direction. In electrothermal mirrors, the response time is governed by thermal convection and conduction through the mirror substrate, ultimately limiting their operation to frequencies typically well below 100 Hz. A similar frequency range applies also to hydraulic mirrors, due to the increased viscosity. For operation above 1 kHz, two actuation technologies prevail: electrostatic and piezoelectric. While most piezoelectric mirrors are typically operated close to their fundamental frequencies, electrostatic mirrors can be designed around thinner and more flexible substrates like SU-8, leading to a nearly flat wide-band response up to their resonance frequencies^12^. Their speed of operation is, however, limited by the squeeze film damping, ubiquitous due to the small air gaps^40,41^. To overcome this fundamental limit, ref. ^12^ studied the backside perforation of the counter electrode. Compared to the lateral air channels reported before^42^, the transient response was improved by a factor of 250, and the vibrational bandwidth of 3 mm diameter SU-8 membranes was extended to 25 kHz. The mirror features so far the highest figure of merit of an electrostatic mirror, operated in air. Recently, ref. ^26^ reported another way to reduce the viscous damping achieved by segmenting the active membrane into several hundred individual actuators with reduced stroke. This way, the interaction between the mirror with its surrounding medium is reduced, allowing a high-speed transient response time corresponding to 15.4 kHz. To further extend the frequency range and completely eliminate the effect of viscous damping ref. ^13^ operated the mirror in a vacuum, reporting a quality factor of 5324 at 460 kHz, which is the fastest varifocal mirror to date (see the top row in Fig. 2). However, due to the resonant operation, this mirror suffers from small useful frequency bandwidth of only 300 Hz. To overcome this, ref. ^11^ exploits a mirror suspension structure exhibiting a hardening nonlinear restoring force, leading to a highly slanted frequency response. This increases the useful *f*_3dB_ bandwidth to 1.1 kHz around 89 kHz. To further increase the frequency bandwidth of resonantly operated electrostatic mirrors ref. ^43^ proposed to use a ring-shaped electrode with a suspended central portion of the membrane across the sealed acoustic cavity. By tuning the length of the acoustic cavity, the mirror amplitude was enhanced by a factor of 4 between acoustical resonance and anti-resonance, while the response could be tuned over a large frequency range between 30 and 40 kHz (Fig. 4c).

### Surface shape fidelity enhancement

**Fig. 5:**
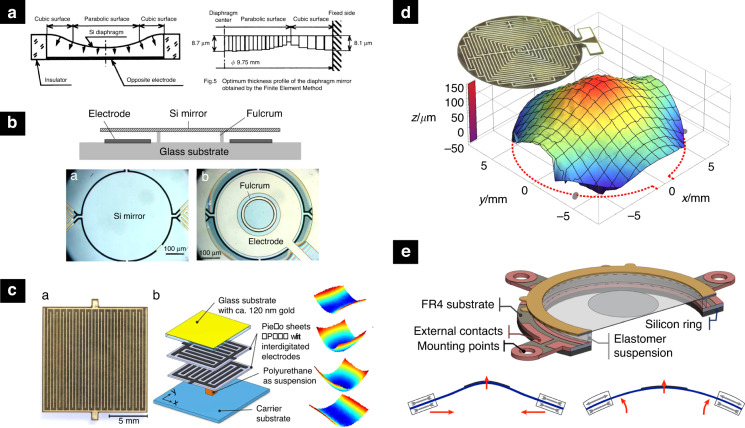
Selected approaches to enhance the shape fidelity. **a** Electrostatic mirror with nonuniform thickness^44^. **b** Actuation scheme to achieve pure bending moment^27^. **c** Active shape control by anisotropic strain using in-plane piezoelectric actuation^53^. **d** Anisotropic strain achieved using actuator optimization achieved in a single plane^39^. **e** Aberration control using double-sided actuation and the flexible substrate^10^. Reprinted with permissions from^10^^,27^^,39^^,44,53^

Increasing the stroke to follow the quest for higher-axial resolution brings more stringent requirements on the shape fidelity to maintain a diffraction-limited depth of focus throughout its focal range. More often than not, dynamic deformation originates from intrinsic geometrical nonlinearities of the warping of the mirror substrate or nonlinearities arising from suspension or the excitation distribution. As such, these effects are more pronounced at larger membrane deformations and hence bring the fundamental trade-off between the stroke and the shape fidelity, introducing practical upper limits on the maximum resolution. These limitations remain to a large extent, not well explored. Nevertheless, several authors tried to improve the shape fidelity both passively, by improving the geometry of the mirror and its suspension mechanism, and actively, by directly controlling the generated pressure distribution. Figure 5 shows a selection of different approaches to enhance the shape fidelity of varifocal mirrors. In a pioneering work from 1995, ref. ^44^ reported a mirror with an edge-clamped membrane featuring nonuniform thickness reduces the negative effect of the support structures. The non-uniformly thick membrane was fabricated by a combination of grayscale lithography and subsequent ion-etching and compared to conventional mirrors with constant thickness, a modulation transfer function with almost two times higher contrast was demonstrated. Most of the later work recognized the need to optimize the mirror’s suspension to attain a better shape fidelity. As such, ref. ^28^ proposed a 10% duty-width segmentation of the outer rim to improve the shape fidelity. Later, Mescheder et al.^45^ studied three different suspensions concerning the deviation from parabolic shape: fixed full membrane, membrane suspended with thin beams, and membrane with a thinned rim. In combination with the ring-shaped electrode, both optimized suspensions were shown to be able to extend the effective useful aperture by a factor of 2. Nakazawa et al.^29^ devised U-shaped cantilevers to weakly support the mirror and improve the shape fidelity by a factor of 5 compared to the edge-clamped design. Hokari & Hane^27^^,^^46^ reported the electrostatic mirror with rotation-free support and electrostatic force applied by the ring electrode around the perimeter of the support to generate pure bending moment and thus achieved less than 4.7 nm deviation from parabola within the 400 μm diameter with a max. stroke of 2.58 μm. In their recent work, ref. ^30^ report the varifocal mirror featuring tangential cantilevers, which allow both in-plane and out-of-plane deformation and thus relax intrinsic stresses, consequentially permitting large out-of-plane deformation at high fidelity.

The second approach to improve the shape fidelity is to control the pressure distribution. Especially in high-amplitude electrostatic mirrors, the electrostatic pressure distribution, being a function of the gap, becomes nonuniform, inducing strong spherical aberration. To circumvent this, Mescheder et al.^45^ proposed a ring-shaped holohedral counter electrode to allow tuning the pressure distribution and achieve a perfect parabolic shape. A more flexible way of controlling the pressure distribution is by segmenting the electrodes into two^28,47,48^ or four segments^9^, allowing the control of the spherical aberration up to their first or third order, respectively. Such control is particularly important in confocal imaging with high numerical apertures^49^. Himmer & Dickensheets^50^ investigated intra-cycle shape at higher frequencies and show that spherical aberration is a function of membrane deflection, indicating that an intra-cycle modulation of relative electrode voltage amplitudes is required to achieve a perfect quadratic shape. Using piezoelectric mirrors, ref. ^11^ used the structured segmented electrodes and demonstrated individual control of defocus and spherical aberrations by tuning the voltage and the excited electrode pattern. There is another aspect concerning aberrations, that arise from off-axis use. If the mirror is illuminated from an angle, significant astigmatism appears in the reflected beam. The first-order astigmatism was shown to be compensated by an elliptical boundary mirror^51,52^. High-order aberrations can be eliminated using piezoelectric actuators. Wapler et al.^10^ reported a bimorph MEMS design using ring-shaped electrodes, applied to both sides of the flexible membrane. Excitation of the electrodes allows both bending and buckling operation and hence control of optical power and spherical aberration. Even better control over the shape of the mirror is allowed by introducing anisotropic strain by lateral excitation relying on the *d*_31_ piezoelectric coefficient. In 2013, Stürmer et al.^53^ reports the use of two laterally excited orthogonal layers of PZT to control the curvature of the mirror in two orthogonal directions and thus simultaneously control optical power and astigmatism. The same group later reported a method for optimizing the interdigitated electrodes to inhomogeneously polarize the piezo layer and hence induce freeform displacement from a single homogeneously polarized active layer. Such an approach provides a new twist to the MOEMS actuators enabling freeform displacements with relatively small bending radii, making it possible to produce novel types of adaptive reflective optics such as rotationally symmetric axicons, hyperbolic sechicons, and non-symmetric pyramicon shapes^39^.

## Applications

In contrast to other focus-tuning technologies, the transition from fundamental scientific research to applications has been rather slow for varifocal MEMS mirrors. One of the main reasons for this is that MEMS mirrors offering high speed and large amplitude focus tuning has not been commercially available until recently. Alternative technologies like TAG, liquid lenses, and liquid crystal lenses seem to be commercially more established and therefore, widely used. Nevertheless, the possible use of varifocal mirrors is broader than presently exploited and may include most of the applications where other focus-tuning technologies are applied today.

### Optical coherence tomography

**Fig. 6:**
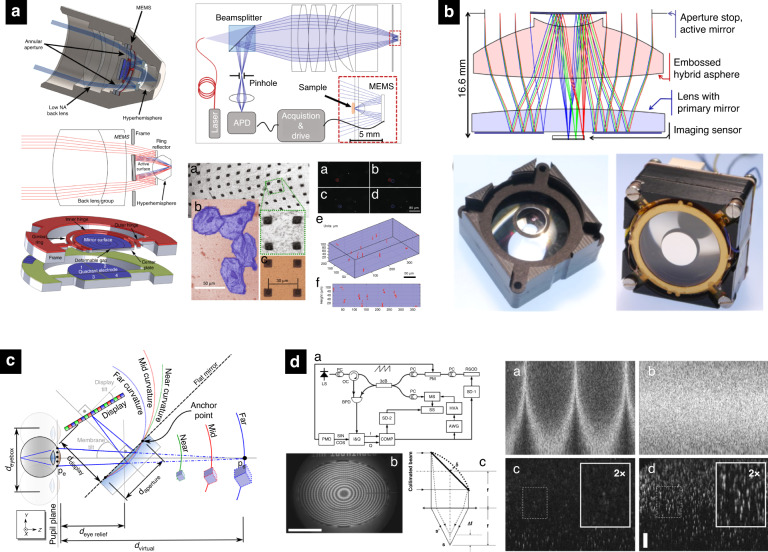
Examples of different applications of varifocal mirrors. **a** Confocal microscopy using MEMS-in-the-lens concept^14^. **b** Catadioptric imaging system^10^. **c** Varifocal augmented reality displays^18^. **d** Varifocal mirror in optical coherence tomography^58^. Reprinted with permissions from^10^^,14,18,58^

Optical coherence tomography (OCT) can provide in-vivo subsurface imaging of tissues with a micrometer resolution to resolve cellular features^54^. MEMS mirrors offer a solution to miniaturize this measurement concept and enable integration into endoscopic probes. These activities have recently been summarized by three comprehensive review papers^47,55,56^. In optical coherence tomography with a fixed focal sample arm arrangement, a compromise between lateral resolution and depth-scanning range has to be faced. Most systems employ low numerical aperture to yield uniform but sub-optimal lateral resolution throughout the entire imaging depth. To improve lateral resolution, ref. ^57^ demonstrated a MEMS mirror to axially shift the focus plane of the sample beam synchronously with the depth-scanning of the coherence gate Fig. 6d. A constant lateral resolution independent of the imaging depth was achieved. The same scheme of dynamic refocusing was later reported in the Doppler OCT to improve imaging performance in resolving microspheres in gel samples and Doppler shift estimation precision in a flow phantom^58^.

### Confocal microscopy

Confocal microscopy relies on axial focus scanning, while back-reflected out-of-focus light is blocked by a pinhole, to allow 3D-sectioning and volumetric imaging of biological samples^55^. Varifocal mirrors are promising miniaturization enablers for these traditionally large scientific devices to allow for in-vivo endoscopic diagnostics. Sasaki & Hane^59^ used an electrostatic varifocal MEMS mirror for a fiber-optic system and demonstrate low-NA confocal sensing. Moghimi et al.^60^ demonstrated a confocal microscope utilizing electrostatically driven varifocal MEMS mirrors. To allow higher numerical apertures of a confocal microscope, ref. ^9^ constructed four-zone varifocal MEMS mirrors to control focus and spherical aberration. Recently, the same group introduced a MEMS-in-the-lens concept (Fig. 6a), where bi-axial scanning is combined with the focus variation into a compact three-axis focusing system, a miniature version of a 3D confocal imaging system demonstrating successful confocal sectioning of polystyrene beads and human cheek cells^14,61,62^.

### High-power laser applications

Their ability to reproduce high-fidelity parabolic surfaces and their lack of dispersion and chromatic aberrations makes the application of varifocal MEMS mirrors especially well suited for laser applications. Future varifocal MEMS mirrors might benefit from a dielectric highly-reflective coating to reduce the losses and increase the ablation threshold. Vdovin & Kiyko^63^ reported the integration of the varifocal mirrors into a laser cavity. Periodic modulation of the mirror induced quick switching between stable and unstable resonator configurations and allowed pulse-period Q-switched generation of 200 W laser. Hydraulic mirrors are a preferred choice for high-power applications, as hydraulic fluid can be used to improve the heat transfer from the membrane and reduce the temperature loading. Rabczuk & Sawczak^64,65^ reported the use of a hydraulic mirror in the laser cavity of the 2 kW laser, and more recently, refs. ^19,36^ reported hydraulic MEMS mirrors into a high-power laser auto-focusing unit for endoscopic laser surgery.

### Incoherent imaging

Little efforts have been expended so far to develop tailored optical systems around these tunable elements to minimize the aberrations for widefield incoherent imaging. Wang et al.^66^ reported a Z-shaped imaging system built around a varifocal mirror and demonstrated auto-focusing abilities. Multiple authors reported the use of varifocal mirrors for auto-focusing and range finding using various different algorithms like deblurring^67^, and entropy-based measure of camera focus^68^. Ishii & Mitsudo^69^ reported a varifocal mirror to acquire focal stacks and a shape-from-focus algorithm to retrieve the 3D shape of solder bumps. Hokari & Hane^27^ demonstrated widefield imaging and noted that aberrations get progressively pronounced where the field of view gets larger than the mirror size. Figure 6b depicts a piezo-based mirror integrated into a Cassegrain-type reflective objective lens, aiming for full-field imaging reported with more than 100 m of focusing^10^. Li et al.^37^ reported optothermal and electrothermal mirrors for full-field imaging, later improved by Paterson et al.^38^ to provide 215 mm of focal range. Kaylor et al.^70^ demonstrated focusing with an electrostatic mirror and has shown to resolve 35 lp/mm at 30% modulation, compared to 68 lp/mm for a perfect optical system with the same aperture.

### Augmented reality

One of the first applications of varifocal mirrors was volumetric stereoscopic 3D displays where a vibrating mirror was synchronized with a 2D display to form an image at different image planes^16,71^. Conceptually similar but way more miniaturized solutions are recently being revived in the context of near-eye displays and augmented reality. A major challenge in such systems is the so-called vergence-accommodation conflict (VAC), where the binocular triangulation distance conflicts with the focusing distance of the eye. Over the years, several solutions to this have been proposed, summarized, and compared in ref. ^5^. Deformable MEMS mirrors have been shown to achieve an accommodation range of 0 to 14 m^−1^ and thus match vergence and stereoscopic retinal disparity to decrease eye fatigue and approximate natural vision^72,73^. Recently, the concept of 3D augmented reality using see-through deformable beamsplitter controlled by air pressure was introduced^18^, see Fig. 6c. Despite the resemblance to the Rawson’s works from 1969, their concept introduces new challenges for varifocal MEMS mirrors such as transparent substrate materials, larger diameters, and large optical powers. Recently, transparent substrates are getting increasing attention in varifocal MEMS to be used as piezoelectric tunable lenses and prisms^74^.

## Outlook

In the future, we might expect an increased prevalence of varifocal mirrors in various optical devices. Microsecond response times make them perfectly suitable for axial scanning in fast laser processing applications for machining curved substrates using enhanced depth of field. The fast operation might further advance the volumetric imaging capabilities in the two-photon microscopy for calcium imaging of neuronal activity^1^. Extremely broad low-GDD reflectance band throughout the focal range qualifies them for multi-wavelength applications like manipulating light in scanning fluorescence microscopy or broadband supercontinuum light and ultrashort laser pulses. Furthermore, the possibility of aberration control makes them well suited for stigmatic diffraction-limited focusing in high NA microscopy^49,75^, while a combination of lateral and axial focusing in a single device offers further miniaturization potential for in-situ endoscopic probes in biomedical diagnostics. The latter is currently already actively pursued using the recently introduced MEMS-in-the-Lens concept^14^.

On top of that, varifocal mirrors are benefiting from highly scalable semiconductor microfabrication technology to cover the future demands in consumer applications. All this makes varifocal mirrors perfectly suited for a number of exciting and innovative applications, where other focus-tuning technologies currently still dominate. In the future, we may expect further substantial improvements in the varifocal technology. Further improvements in the operation speeds of varifocal mirrors towards the MHz regime are likely to open new questions concerning fatigue and lifetime that are related to the intrinsic limits of materials. There still is potential to increase the stroke of varifocal MEMS mirrors and hence the axial resolution. Wafer-level vacuum packaging capabilities are recently getting within reach of MEMS fabs and reduction of viscous damping is expected to further boost the quality factors, potentially achieving 1000 distinctly resolvable planes in the near future. To maintain a diffraction-limited performance throughout the entire focal range, not only the stroke but also the shape fidelity will have to be further improved. Intra-cycle active control, inverse design^76^, or highly selective modal excitation^77^ could support the maintenance of diffraction-limited performance. Optical system designs optimized specifically around tunable reflective membranes are expected to further improve imaging performances. A combination of metasurfaces^78^ and diffractive optical elements or thin-film optical filters directly into the thin-film membranes^79^ might find new ways towards compact advanced optical sensing.

## Conclusion

Varifocal mirrors have gone through a remarkable evolution process in the last five decades. In this review, the past progress is summarized and reported varifocal MEMS devices from the literature are compared by a figure of-merit, proportional to the scanning speed and the axial resolution. Various approaches on how to enhance axial resolution, speed, and shape fidelity are discussed. While the study showed that electrostatically and piezoelectrically actuated varifocal mirrors outperform other actuation technologies, pneumatic, hydraulic, and thermal mirrors lower the entry barrier to build them, potentially reaching researchers from other fields. We therefore appreciate the diversity of designs and actuation mechanisms and encourage further research toward improved, optimized transduction mechanisms. Recent progress of the varifocal technology is showing promise to reach 1000 resolvable planes and ultrafast operation with sub-microsecond response times. Today, there is a high demand for miniaturized, cost-effective, and performant axial scanning devices for applications such as real-time volumetric neural activity imaging, laser processing, augmented reality, and biomedical diagnostic. These demands, in combination with the scalability and maturity of MEMS fabrication processes, establish a fruitful environment for the next remarkable half-century of technological development and application of varifocal MEMS mirrors.

## Appendix

**Table 1:** Varifocal mirror data reported in the literature and depicted in Fig. 2

Authors	Year	Actuation	*f*/Hz	shape	*d*_1_/mm	*d*_2_/mm	Δ*O**P**R*/m^−1^	*F**O**M*/mHz
Fujita et al.^80^	1988	es	0	rectangular	14.8	14.8	1.8	-
Hisanaga et al.^81^	1993	es	0	circular	10.0	10.0	10.8	-
Burns & Bright^82^	1998	et	120	circular	1.0	1.0	65.7	7.9e-03
Divoux et al.^83^	1998	em	0	circular	30.0	30.0	0.2	-
Himmer et al.^28^	2001	es	$\underline{25000}$	circular	1.0	1.0	24.9	6.2e-01
Vdovin & Kiyko^63^	2001	es	250	circular	10.0	10.0	0.5	1.2e-02
Mescher et al.^84^	2002	pe	0	circular	0.6	0.6	45.9	-
Mescher et al.^84^	2002	pe	0	circular	0.3	0.3	107.9	-
Friese et al.^85^	2003	es	0	circular	1.0	1.0	72.0	-
Rabczuk & Sawczak^64^	2003	hy	0	circular	25.0	25.0	0.3	-
Himmer & Dickensheets^51^	2004	es	10000	circular	1.0	1.0	32.0	3.2e-01
Qi et al.^57^	2004	es	$\underline{8000}$	elliptical	1.4	1.0	16.7	1.3e-01
Rabczuk & Sawczak^65^	2004	hy	0	circular	64.0	64.0	0.2	-
Shao et al.^48^	2004	es	0	circular	0.7	0.7	100.0	-
Friese & Zappe^86^	2005	es	0	circular	5.0	5.0	24.3	-
Ishii & Mitsudo^87^	2005	pe	20	circular	6.0	6.0	17.8	1.3e-02
Mi et al.^88^	2005	es	0	circular	0.5	0.5	90.3	-
Bonora & Poletto^33^	2006	es	0	circular	10.0	10.0	0.4	-
Dickensheets et al.^89^	2006	es	10000	circular	1.2	1.2	49.3	7.7e-01
Mescheder et al.^45^	2006	es	0	circular	5.0	5.0	10.0	-
Rabczuk & Sawczak^87^	2006	pe	0	circular	25.0	25.0	0.2	-
Tanaka et al.^90^	2006	pe	100	rectangular	12.0	12.0	0.0	2.9e-04
Yang et al.^58^	2006	es	8000	elliptical	1.4	1.0	38.4	3.1e-01
Yeh et al.^91^	2006	pn	0	circular	4.0	4.0	57.0	-
Kanno et al.^92^	2007	pe	300	circular	8.0	8.0	0.7	1.4e-02
Alzaydi et al.^93^	2008	hy	15	circular	0.9	0.9	275.7	3.7e-03
Friese & Zappe^94^	2008	es	5	circular	7.0	7.0	3.9	9.6e-04
Aoki et al.^95^	2009	pe	0	elliptical	5.0	3.8	6.4	-
Hokari & Hane^27^	2009	es	0	circular	1.5	1.5	41.7	-
Hokari & Hane^27^	2009	es	0	rectangular	1.0	0.4	29.9	-
Lukes et al.^25^	2009	es	0	circular	2.0	2.0	33.2	-
Wang et al.^66^	2009	es	2500	circular	4.5	4.5	20.0	1.0e+00
Hsieh et al.^52^	2010	es	0	circular	3.0	3.0	21.3	-
Kaylor et al.^70^	2010	es	0	circular	3.0	3.0	6.5	-
Lutzenburger et al.^96^	2010	es	0	circular	3.0	3.0	21.3	-
Sadeghi et al.^35^	2010	es-hy	15	circular	2.2	2.2	35.2	2.6e-03
Hashizume et al.^97^	2011	em	0	circular	14.0	14.0	0.2	-
Ma et al.^98^	2011	pe	0	circular	35.0	35.0	0.1	-
Moghimi^42^	2011	es	100	circular	3.0	3.0	21.3	1.9e-02
Sasaki & Hane^31^	2011	es	0	circular	0.4	0.4	11.9	-
Lukes & Dickensheets^99^	2012	es	0	elliptical	4.2	3.0	6.0	-
Moghimi et al.^60^	2012	es	0	circular	2.0	2.0	32.0	-
Sasaki & Hane^59^	2012	es	0	circular	0.3	0.3	18.5	-
Li et al.^37^	2013	et	8	circular	1.2	1.2	18.0	2.2e-04
Li et al.^37^	2013	ot	8	circular	1.2	1.2	28.0	3.3e-04
Lukes & Dickensheets^100^	2013	es	2800	elliptical	4.2	3.0	13.2	3.3e-01
Moghimi et al.^12^	2013	es	20000	circular	3.0	3.0	17.8	3.2e+00
Sasaki et al.^101^	2013	es	$\underline{21000}$	circular	1.0	1.0	12.3	2.6e-01
Strathman et al.^102^	2013	pe	216	circular	0.8	0.8	40.0	5.5e-03
Stürmer et al.^53^	2013	pe	0	rectangular	14.0	14.0	0.1	-
Wapler et al.^39^	2013	pe	0	circular	15.0	15.0	0.3	-
Wapler et al.^39^	2013	pe	0	circular	15.0	15.0	5.7	-
Wapler et al.^39^	2013	pe	0	circular	15.0	15.0	4.6	-
Wapler et al.^39^	2013	pe	0	circular	15.0	15.0	4.6	-
Wapler et al.^39^	2013	pe	0	circular	15.0	15.0	1.4	-
Wapler et al.^39^	2013	pe	0	circular	15.0	15.0	28.4	-
Wapler et al.^39^	2013	pe	0	circular	15.0	15.0	1.8	-
Wu et al.^103^	2013	es	1000	rectangular	1.9	1.9	16.4	5.9e-02
Wu et al.^103^	2013	es	1000	rectangular	1.9	1.9	33.7	1.2e-01
Hossain et al.^20^	2015	em-pn	0	circular	5.0	5.0	20.0	-
Lukes^104^	2015	es	0	circular	5.0	5.0	9.5	-
Moghimi & Dickensheets^34^	2015	es-pn	0	circular	5.0	5.0	12.8	-
Morrison et al.^32^	2015	et	200	circular	0.4	0.4	2131.8	6.8e-02
Paterson et al.^38^	2015	et	0	circular	1.2	1.2	39.4	-
Liu & Dickensheets^61^	2016	es	2800	circular	4.0	4.0	16.0	7.2e-01
Lukes et al.^9^	2016	es	0	circular	4.0	4.0	12.0	-
Nakazawa et al.^29^	2016	es	$\underline{9500}$	circular	2.0	2.0	4.3	1.6e-01
Kopf et al.^105^	2017	pe	0	circular	25.0	25.0	0.5	-
Nakazawa et al.^106^	2017	es	0	circular	2.0	2.0	6.5	-
Nakazawa et al.^106^	2017	es	$\underline{18300}$	circular	2.0	2.0	9.1	6.7e-01
Geraldes et al.^19^	2018	hy	6	circular	4.0	4.0	79.5	7.0e-03
Janin et al.^107^	2018	pe	$\underline{26100}$	circular	1.4	1.4	14.2	7.3e-01
Janin et al.^107^	2018	pe	$\underline{107000}$	circular	1.4	1.4	7.0	1.5e+00
Liu et al.^62^	2018	es	0	circular	4.0	4.0	9.0	-
Wapler et al.^10^	2018	pe	1750	circular	10.0	10.0	16.0	2.8e+00
Geraldes et al.^36^	2019	hy	0	elliptical	4.2	3.0	50.2	-
Liu et al.^14^	2019	es	0	circular	4.0	4.0	9.1	-
Ersumo et al.^26^	2020	es	15440	circular	8.2	8.2	2.4	2.5e+00
Sasaki et al.^13^	2020	es	$\underline{290130}$	circular	1.0	1.0	38.0	1.1e+01
Sasaki et al.^13^	2020	es	$\underline{462730}$	circular	1.0	1.0	71.4	3.3e+01
Kallmann et al.^108^	2021	es	0	rectangular	8.0	2.0	5.8	-
Mescheder et al.^30^	2021	es	0	circular	5.0	5.0	5.1	-
Pribošek et al.^11^	2021	pe	$\underline{90000}$	circular	2.6	2.6	205.9	1.3e+02

The data were chronologically sorted. Resonantly operated mirrors are marked by underlined f/Hz values

*es* electrostatic, *pe* piezoelectric, *hy* hydraulic, *pn* pneumatic, *et* electrothermal, *ot* optothermal, *em* electromagnetic, *es-pn* electrostatic-pneumatic, *em-pn* electromagnetic-pneumatic, *es-hy* electrostatic-hydraulic

### Axial resolution

The physical thickness of one resolvable plane in the axial direction Δ*z*_Rayleigh_ can be described by the Rayleigh criterion (Equation (3)), where *z* is the position in the axial direction *r* is the mirror radius and *λ* is the light wavelength.

3 $${{\Delta }}{z}_{{\mathrm{Rayleigh}}}=\frac{2\lambda {z}^{2}}{{r}^{2}}$$

The mirror sag *s* associated with this position *z* in the axial direction and its derivative are given in Equation (4). This relationship is the same approximation as in Fig. 1.

4 $$s=\frac{{r}^{2}}{4z}\,\,,\,\,\frac{ds}{dz}=\frac{-{r}^{2}}{4{z}^{2}}$$

As shown in Equation (5), the number of resolvable planes *N*_planes_ is computed by integrating the reciprocal of Δ*z*_Rayleigh_ from the minimal achievable focal length *z*_min_ to the maximal achievable focal length *z*_max_ .

5 $${N}_{{\mathrm{planes}}}=\int\nolimits_{{z}_{{\mathrm{min}}}}^{{z}_{{\mathrm{max}}}}\frac{1}{{{\Delta }}{z}_{{\mathrm{Rayleigh}}}}dz=\int\nolimits_{{\delta }_{m,{\mathrm{max}}}}^{{\delta }_{m,{\mathrm{min}}}}\frac{1}{{{\Delta }}{z}_{{\mathrm{Rayleigh}}}}{\left(\frac{ds}{dz}\right)}^{-1}ds$$

After substituting *z* for *s* according to Equation (4), the integral can be calculated. The result in Equation (6) shows that the number of resolvable planes in the axial direction only depends on the stroke Δ*s* and the wavelength *λ*.

6 $${N}_{{\mathrm{planes}}}=\int\nolimits_{{\delta }_{m,{\mathrm{max}}}}^{{\delta }_{m,{\mathrm{min}}}}\frac{{r}^{2}}{2\lambda {z}^{2}}\frac{-4{z}^{2}}{{r}^{2}}ds=\int\nolimits_{{\delta }_{m,{\mathrm{max}}}}^{{\delta }_{m,{\mathrm{min}}}}\frac{-2}{\lambda }ds=\frac{2}{\lambda }{{\Delta }}s$$
